# Hemostasis of idiopathic recurrent epistaxis in children with microwave ablation: a prospective pilot case series

**DOI:** 10.1186/s40463-019-0393-0

**Published:** 2019-12-18

**Authors:** Zheng-cai Lou

**Affiliations:** Department of Otorhinolaryngology, YiWu central Hospital, 699 Jiangdong Road, Yiwu city, Zhejiang, 322000 China

**Keywords:** Anterior epistaxis, Children, Microwave ablation, Safety, Side effect

## Abstract

**Objective:**

We evaluated microwave ablation (MWA) for treatment of idiopathic recurrent anterior epistaxis (RAE) in children, in terms of technical feasibility, efficacy, and safety.

**Study design:**

A prospective pilot case series.

**Setting:**

Tertiary university hospital.

**Methods:**

Children with idiopathic RAE were treated with endoscopic MWA and reevaluated at 1 and 4 weeks and at 6 months thereafter. The primary outcome was successful hemostasis on the day of the procedure. Secondary outcomes were the rebleeding rates after 1 and 4 weeks, and 6 months, and any complications.

**Results:**

Of the 92 children with idiopathic RAE who met the inclusion criteria, the operation was interrupted in 7 children due to intraoperative noncooperation, and silver nitrate cautery was performed instead. All procedures were completed, and hemostasis was achieved within 10–20 s by MWA in 85 children. Two to four ablations were conducted. No recurrent epistaxis occurred and no severe MWA -related complications, such as septal perforation or synechiae formation, were observed at the 6-month follow-up.

**Conclusions:**

Endoscopic MWA is a feasible and safe technique for the treatment of idiopathic pediatric RAE in the clinical setting, especially those cases that do not respond to in-office chemical cautery.

## Introduction

Idiopathic epistaxis is a common complaint seen in children in rhinology outpatient clinics. In most pediatric cases, idiopathic epistaxis originates from Kiesselbach’s plexus, which is located in the anteroinferior portion of the nasal septum; thus, this condition is also known as recurrent anterior epistaxis (RAE) [1, 2]. The ideal treatment for idiopathic RAE has yet to be elucidated. Most cases are self-limiting or resolve with simple measures, such as pinching of the nose or hydration of the nasal mucosa with saline solution. Although silver nitrate cautery is the most common method of chemical cautery for the treatment of RAE, it has mainly been used to control bleeding associated with small vessels and ulceration of the nasal mucosa [3]. Electrosurgery appears to be more effective than silver nitrate in controlling bleeding telangiectasias [4]. Johnson et al. [1] suggested that bipolar electrocautery may be a superior treatment in children with RAE at risk of severe bleeding, in whom chemical cautery will likely fail. However, conventional electrosurgery produces high temperatures (exceeding 400 °C) [5] and forms a secondary crusting, resulting in local pain intraoperatively and recurrent epistaxis [6].

Microwave ablation (MWA) is a new minimally invasive therapeutic technique that can cause high tissue temperatures of 60–100 °C and covers a large tissue volume, thus allowing for rapid ablation and a large area of coagulation [7, 8]. Many studies have shown that MWA is safe and effective in the treatment of active hemorrhage of the liver [9–11] and epithelioid hemangioma [12]; its use can minimize intraoperative blood loss. MWA has been used to treat severe epistaxis in adult patients in China, and has shown high success rates with minimal side effects [13, 14]. However, no studies have evaluated the efficacy and safety of MWA for the treatment of idiopathic RAE in children. This study was performed to evaluate the technical feasibility, efficacy, and safety of MWA in children with idiopathic RAE seen in the outpatient clinic.

## Materials and methods

### Ethical considerations

This study was approved by the Institutional Ethical Review Board of Yiwu Central Hospital (Yiwu, Zhejiang, China). Informed consent was obtained from all study participants or their parents.

The study population was recruited from children diagnosed with unilateral idiopathic RAE who visited the Department of Otorhinolaryngology, Yiwu Central Hospital, between February 2016 and January 2019. The inclusion criteria were: (1) A history of repeated epistaxis with at least four episodes, at least one episode a week during the preceding 4 weeks, and frequent episodes within the last 2 weeks; (2) age ≥ 8 years, failure of topical treatment with an antiseptic ointment, with and without silver nitrate cautery, prior to MWA; (3) a Katsanis epistaxis scoring system (ESS) score of 7–10 [15]; and (4) bleeding originating from Kiesselbach’s plexus, located in the anteroinferior portion of the nasal septum, with the bleeding point showing an isolated mucosal bulge (primary telangiectasia, or an isolated mucosal bulge with a red or white top) but not ulceration of the nasal mucosa.

Preoperative laboratory investigations were performed in all patients to exclude bleeding or coagulation disorders. In 1988, Katsanis et al. developed an ESS based on five components of the epistaxis history, assigning a score of 0–2 to each component. The scores from each of the five components were then summed, yielding a total score for each child. Children with a summed score between 0 and 6 were classified as having mild epistaxis and those with a summed score between 7 and 10 as having severe epistaxis. Only those with severe epistaxis were treated with nasal cauterization [15]. The Katsanis ESS was administered prior to MWA. Treatment of idiopathic RAE for which no specific cause has been identified involves repeated nasal bleeding in patients aged up to 18 years.

### Technique

All children were placed in a sitting position and hemostasis was completed in a clinical setting under local anesthesia. Blood in the nasal cavity was removed by suction. Cotton wool pledgets soaked in 1% lidocaine and 0.1% adrenaline were placed in the common meatus for 5 min three times, for a total placement time of 15 min. The nasal cavity was examined endoscopically to identify the bleeding point and exclude posterior epistaxis.

**Fig. 1:**
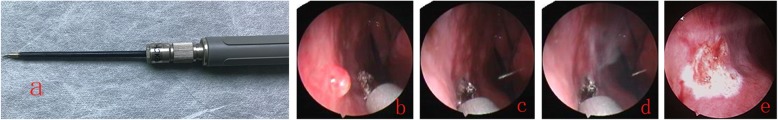
(**a**) Microwave ablation (MWA) antenna with a split-type double needle. (**b**) A lesion resembling capillary hemangioma. (**c, d**) MWA coagulation. (**e**) The ablation zone

An MWA device (EBH-IV; Zhuhai Hokai Medical Instruments Co., Ltd., Zhuhai, China) with a 2450 MHz cooled-shaft contact-type antenna was used to ablate the bleeding point in the ENT Treatment Room, with output power of 50 watts. The microwave antenna was 9 cm in length and 3 mm in outer diameter, and the length of the exposed and non-insulated antenna tip was 5 mm. The size of the MWA antenna was similar to the outer diameter of the bipolar cautery or monopolar suction device, allowing it to easily reach the anteroinferior portion of the nasal septum under endoscope guidance (Fig. 1). The tip of the antenna was a split-type double needle. The double needle has a width of 1 mm and the length of the upper part of the antenna tip is 2 mm. The upper part of the antenna tip was bought into contact with the nasal mucosa or bleeding point. A footplate-operated switch was used to control the ablation time, and the length, width, and depth of penetration of the thermal lesion. The microwave stopped ablating immediately on deactivation of the footplate-operated switch. The lesions were ablated in a distal-to-proximal direction to achieve a uniform gray-white ablation zone in the lesion and surrounding tissue (Fig. 1). The microwave application time was 1–3 s for each ablation treatment, and repeated ablation could be performed for different areas on the same lesion and adjacent nasal mucosa; however, multiple ablations in the same area should be avoided to avoid septal perforation. All procedures were performed by a single surgeon. Standard postoperative care included the topical application of an antibiotic ointment and nasal irrigation.

The children were reevaluated, and the nasal cavity reexamined, at 1 and 4 weeks and 6 months after MWA. The primary outcome was successful hemostasis on the day of the procedure. Secondary outcomes were the rebleeding rates at 1 and 4 weeks, and 6 months, and any complications (postoperative crust formation, synechiae formation, or septal perforation). Because of large variation in the degree of bleeding reported by the patients or their parents, in this study a bleeding event following ablation at the same site, confirmed by any doctor and requiring intervention, was considered as recurrent epistaxis during the follow-up period. Interventions included additional silver nitrate cautery, reablation, and nasal packing.

## Results

Of the 93 children who fulfilled the inclusion criteria, 1 child was excluded due to hemophilia. Thus, the study population consisted of 92 children (64 boys and 28 girls) with idiopathic RAE. All of the children had unilateral epistaxis in which the bleeding points were located in the anteroinferior portion of the nasal septum. The demographic data of the 92 children with idiopathic recurrent epistaxis are shown in Table 1. The bleeding point consisted of an isolated mucosal bulge with a red or white top in 36 (39.1%) patients and isolated primary telangiectasias in 56 (60.9%) patients. The mean ESS score of the 92 patients was 7.86 ± 0.59 and the mean patient age was 14.7 ± 1.3 years (range: 8–18 years). The left nasal cavity was involved in 59 (64.1%) patients and the right nasal cavity in 33 (35.9%) patients. The average duration of epistaxis was 2.2 ± 0.6 weeks. A total of 37 (40.2%) children had a history of silver nitrate cautery.

**Table 1:** Demographic data of idiopathic recurrent epistaxis in 92 children

Variable	n (%)
Gender	
Male	64 (69.6%)
Female	28 (30.4%)
Age (years)	
Mean ± SD	14.7 ± 1.3
Minimum	8
Maximum	18
The side of nasal cavity	
Left side	59 (64.1%)
Right side	33 (35.9%)
The bleeding point	
Mucosal bulge with a red or white top	36 (39.1%)
Primary telangiectasias	56 (60.9%)
Average duration of epistaxis (weeks)	2.2 ± 0.6
Prior silver nitrate attempts	37 (40.2%)
Katsanis ESS	
Mean ± SD	7.86 ± 0.59
7	80 (87.0%)
8	11 (12.0%)
9	1 (1.0%)

All procedures were done in a clinical setting under local anesthesia. The operation was interrupted in seven (7.6%) patients due to intraoperative noncooperation and the procedure was changed to silver nitrate cautery. Of the seven (7.6%) patients, two were afraid of the endoscope and microwave antenna, and five could not tolerate the intraoperative pain.

Of the seven patients with failed MWA, four underwent silver nitrate cautery twice, two patients were treated with silver nitrate cautery and anterior nasal packing with a gelatin sponge four times, and one patient received anterior nasal packing with Merocel.

**Fig. 2:**
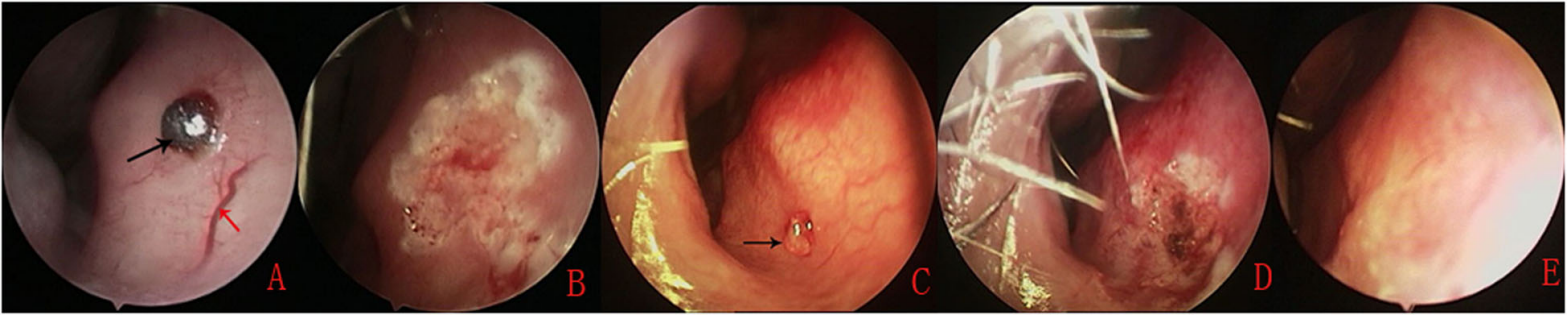
An isolated mucosal bulge with a red top located in the anteroinferior portion of the nasal septum (**a**) was subsequently ablated by MWA (**b**). An isolated mucosal bulge with a white top located in the anteroinferior portion of the nasal septum (**c**) was subsequently ablated by MWA (**d**). One week after ablation (**e**). Black arrows indicate the isolated mucosal bulge; red arrows indicate prominent vessels

All the procedures were completed and hemostasis was achieved within 10–20 s by MWA in 85 (92.4%) children (Fig. 2). Two to four ablations were conducted. Of the 85 (92.4%) patients, 2 (2.4%) had minor postablation errhysis that did not require further treatment, and 7 (8.2%) had short postablation self-resolving bleeds that did not require treatment by a doctor. The cost of MWA was 79 RMB (approximately US$13.17), and the MWA antenna was reusable.

All of the children were discharged after ablation, and all were monitored at each follow-up point. At the 1-week follow-up, no black crusting was observed in the ablation zone in 85 children, while a gray-white pseudomembrane covered the ablation zone in 11 (12.9%) children. The ablation zone showed normal nasal mucosal morphology in all cases at 4 weeks after MWA treatment. In addition, no recurrent epistaxis occurred and no severe MWA-related complications, such as septal perforation or synechiae formation, were observed at the 6-month follow-up.

## Discussion

Idiopathic RAE in childhood is encountered frequently in outpatient clinics. The most common treatments for idiopathic RAE are antiseptic ointment, chemical cauterization, and nasal packing. However, the optimal method for managing children with anterior epistaxis has yet to be determined and further research is thus required. In children with persistent epistaxis in whom first-aid measures have failed and a bleeding vessel is visible, silver nitrate cautery is the treatment of choice [16]. However, some children continue to suffer from epistaxis despite nasal cautery. Felek et al. [3] reported the complete or nearly complete control of only 76% of RAE with superficial vessels or a network of small vessels treated with one round of silver nitrate cauterization, and of 86% of RAE after the second cauterization, based on a mean follow-up period of 8 months. In a previous study [17], complete resolution of RAE after one round of silver nitrate cautery was achieved in only 77% (33/43) of the cases and after a second round of cauterization in 84% (36/43), after a mean follow-up of 4 months. Thus, if chemical cautery with silver nitrate does not sufficiently stop the bleeding in children with idiopathic recurrent recalcitrant epistaxis, electrocautery may be necessary [1, 18]. However, electrocautery forms a secondary crust at the site where the heated probe adhered to the tissue coagulum, causing rebleeding upon removal of the probe [6], thus accounting for a recurrent bleeding rate of 8–8.5% [1, 19].

MWA was reported to show excellent hemostasis results in cases of active hemorrhage of the liver, epithelioid hemangioma, and hemorrhaging angiosarcoma [9–12]. MWA has also been used to treat severe epistaxis in adult patients, and it only requires a short ablation time and achieves a high hemostasis rate with minimal complications [13, 14]. In MWA, the microwave antenna in the target tissue quickly reaches a high temperature, resulting in the rapid induction of coagulation necrosis and therefore hemostasis [10]. We examined the utility of MWA for treating idiopathic RAE in children. The main patient selection criterion was an isolated mucosal bulge or isolated primary telangiectasias involving the anterior septum, chemical cauterization of which may worsen bleeding or necessitate repeated cautery and nasal packing. In this study, only 7 of 92 patients were unable to tolerate MWA treatment, while all the procedures were completed and hemostasis was achieved within 10–20 s by MWA in 85 children. Because the use of MWA enables both lesion removal and hemostasis with the same instrument, MWA is also effective for active bleeding and shortens the hemostasis time. The short procedure time was well tolerated by most of the children in our study and the operation could be completed successfully. In addition, there were no cases of recurrent epistaxis over the 6-month follow-up period. This may have been due to the fact that an endoscope was used to precisely identify and then ablate the bleeding point as well as the feeding vessels. The resulting scarring and fibrosis in the perichondrium of the septum reduced the possibility of neovascularization and recurrent bleeding. An animal study performed by Dano et al. [20] showed that fibrosis and the reduced vascularity of the nasal septal mucosa prevent future episodes of epistaxis. Levi et al. [18] proposed that septoplasty without removal of the septal cartilage was efficacious in treating recurrent recalcitrant pediatric epistaxis because of the fibrosis and scarring of the causative vasculature on the mucosal flaps.

Assessment of the tolerability and safety of MWA under local for on anterior epistaxis is crucial to avoid adverse effects in children. In this study, the minimum age of the patients was 8 years, and 92.4% (85/92) of them tolerated the operation. This high tolerance rate was related to the following factors. First, appropriate cases were selected, and sufficient anesthesia was achieved, so most of the children were cooperative. Second, MWA only produced an average temperature of 65–100 °C, which was significantly lower than the temperatures associated with bipolar cautery, which exceed 400 °C [3, 8]; thus, there was less intraoperative pain. Our previous study suggested that the thermal lesions induced by MWA were approximately 2 mm in length and 1 mm in width, occurring at a depth of approximately 0.5–1 mm [11]. Use of an endoscope may allow more effective visualization of the bleeding point and perichondrium of the anterior nasal septum. Given the short ablation time and small thermal lesions, septal perforation was unlikely. Nevertheless, multiple ablations at the same septal cartilage site should be avoided.

## Conclusions

In a clinical setting, MWA under endoscopic guidance is a feasible and safe technique for the treatment of pediatric idiopathic RAE characterized by an isolated mucosal bulge or isolated primary telangiectasias. It is especially effective in older children in whom in-office chemical cautery has already failed. MWA is a simple, inexpensive and minimally invasive treatment method, which provided rapid hemostasis in children with idiopathic RAE.

## Data Availability

The datasets supporting the conclusions of this article are included within the article.

## Abbreviations

MWA: Microwave ablation
RAE: Recurrent anterior epistaxis
